# Clear-cell carcinoma originating from cesarean section scar: two case reports 

**DOI:** 10.1186/s13256-021-02775-9

**Published:** 2021-04-03

**Authors:** Seyedeh Razieh Hashemi, Mahdi Morshedi, Houshyar Maghsoudi, Arezoo Esmailzadeh, Ibrahim Alkatout

**Affiliations:** 1 Department of Obstetrics and Gynecology, Baqiyatallah University of Medical Sciences, Tehran, Iran; 2 Department of Surgery, Trauma Research Center, Baqiyatallah University of Medical Sciences, Tehran, Iran; 3 Department of Radiology, Baqiyatallah University of Medical Sciences, Tehran, Iran; 4 Endometriosis Research Center, Iran University of Medical Sciences (IUMS), Tehran, Iran; 5 University Hospital Schleswig-Holstein, Campus Kiel, Kiel School of Gynaecological Endoscopy, Arnold-Heller-Str. 3, Haus 24, 24105 Kiel, Germany

**Keywords:** Clear-cell carcinoma, Endometriosis, Abdominal wall, Surgical site, Cesarean section scar

## Abstract

**Background:**

Clear-cell carcinoma arising from the surgical cesarean section scar is very infrequent. The present study reports two patients with clear-cell carcinoma arising from an abdominal wall scar 20 and 23 years after their last cesarean section.

**Case presentation:**

Both Iranian patients had prior cesarean sections nearly 20 years earlier. Patients 1 and 2 had transverse and vertical abdominal incisions, respectively. The initial clinical presentation was a huge lower abdominal mass at the site of the previous cesarean section scar. Both patients underwent abdominal wall mass biopsy. The histological analysis revealed the presence of malignancy. Both patients underwent full-thickness resection of the abdominal wall mass. All surgical margins were tumor-free; however, patient 1 had a very narrow tumor-free margin near the pubic symphysis. As the imaging report of patient 2 revealed the presence of a pelvic mass, the exploration of the intraperitoneal space, simple total abdominal hysterectomy (TAH), bilateral salpingo-oophorectomy (BSO), and the excision of enlarged pelvic lymph nodes were performed during the surgery. Six cycles of paclitaxel and carboplatin every 3 weeks as adjuvant chemotherapy was administered for both patients after the surgery. One of the patients had disease recurrence 5 months after the termination of chemotherapy, and the other is still disease-free. These two patients had similar pathology and received a similar initial adjuvant treatment; however, they were different in terms of the direction of tumor spread, tumor distance from the pubic symphysis, status of tumor margins, and surgical procedures.

**Conclusions:**

We encountered distinct prognoses in the clear-cell carcinoma of cesarean section scars presented herein. The researchers can recommend complete surgical excision of the abdominal wall mass with wide tumor-free margins, exploration of the abdominopelvic space, TAH, and BSO during the first surgery.

## Background

Endometriosis is one of the most frequent conditions in reproductive-aged women, with a prevalence rate of at least 10–15% [1]. Endometriosis has been defined as the abnormal growth of endometrial tissues outside the uterus. Abdominal wall endometriosis (AWE) has typically been observed in patients undergoing gynecological surgeries such as caesarian section and hysterectomy [2, 3]. Malignant transformation of extra-ovarian endometriosis is infrequent [4, 5]. The risk of the transformation of endometriosis to a malignant disease is currently estimated to be 0.3–1.0% [6]. Clear-cell carcinoma is the second most frequent histological variant, and endometrioid carcinoma [7]. Abdominal wall clear-cell carcinoma is an extremely rare event. Following the documentation of the first case in 1986 [8], only a few cases have been reported to date. Based on the published studies in the literature, the time lag from the first surgery to the diagnosis of malignancy is 6–20 years [4, 5]. The management of abdominal wall clear-cell carcinoma has not been well attended to because this tumor is extremely rare and the published literature is not rich in this regard. We report two patients with clear-cell carcinoma in the lower abdominal wall at the site of their previous cesarean section scar. Patient 1 had a history of abdominal cesarean scar endometriosis before menopause; however, we did not have any records for the endometriosis of patient 2. Standard written informed consent was obtained from both patients to use data and images for research and publication purposes.

## Case presentation

### Patient 1

**Fig. 1:**
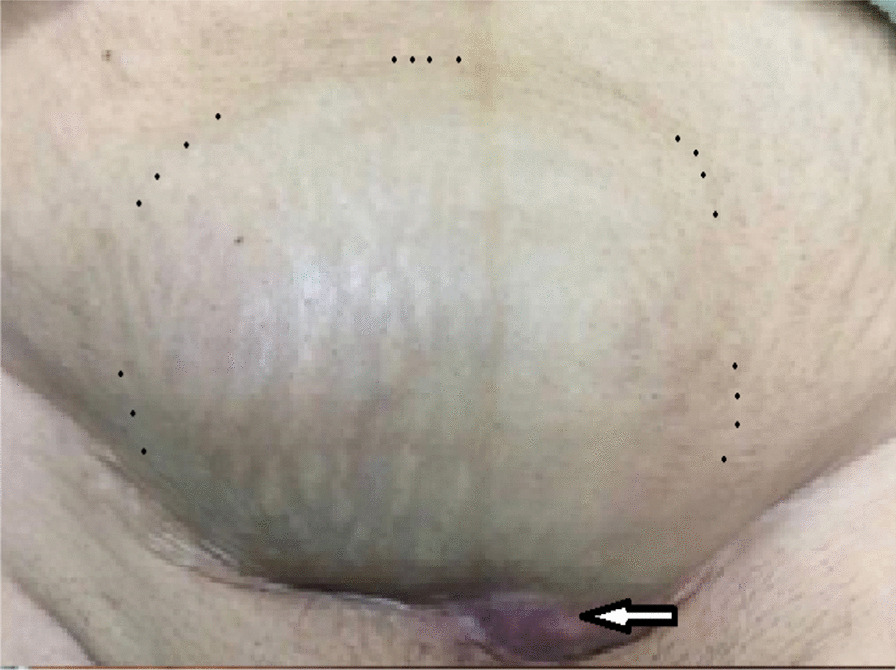
A huge mass originating from the horizontal cesarean scar in patient. Black spots show the margins of the mass. The site of previous orifice is marked by an arrow. Pfannenstiel scars are very close to the pubic symphysis

**Fig. 2:**
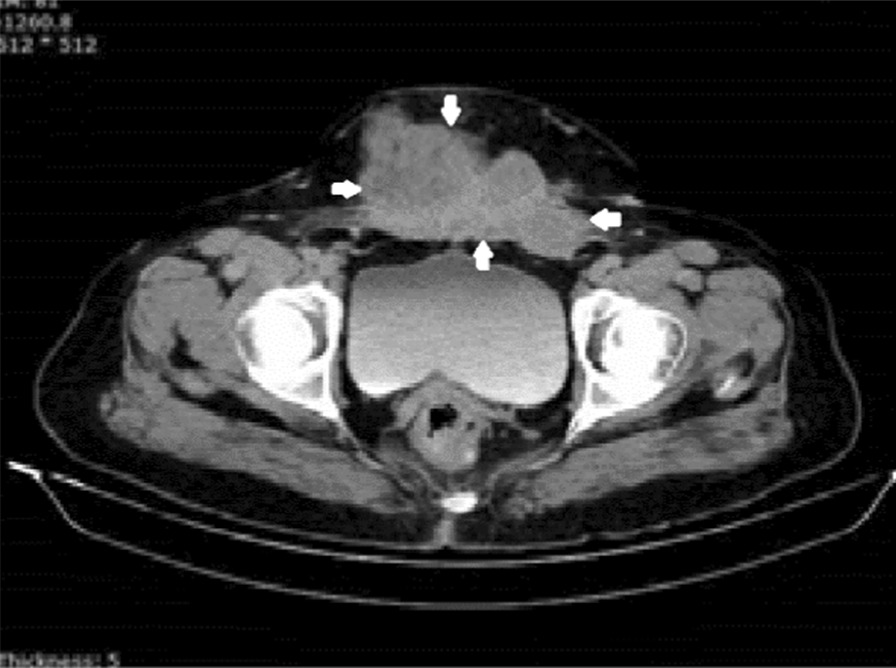
Preoperative pelvic computed tomography scan of patient 1. Borders of the abdominal wall mass are marked by arrows

**Fig. 3:**
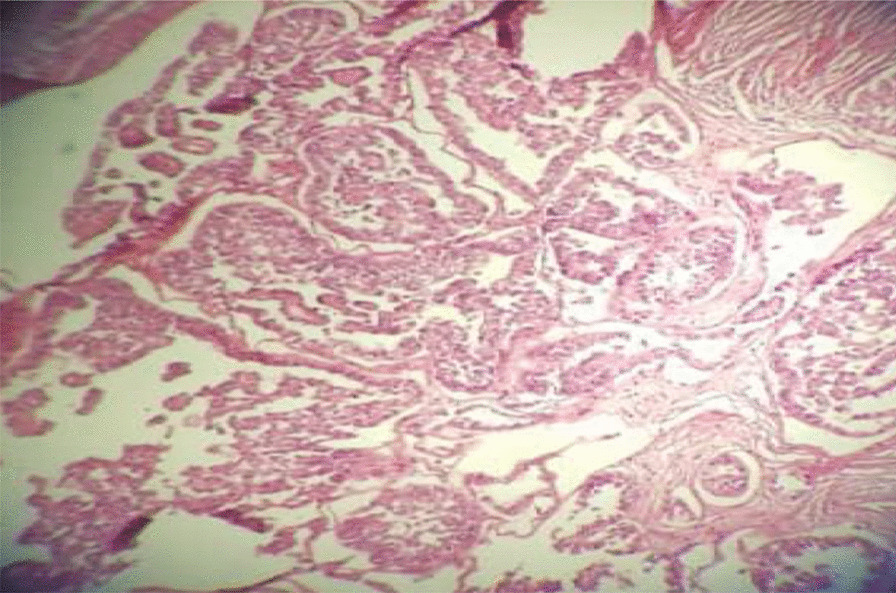
Clear-cell carcinoma of the abdominal scar. Malignant glands are lined by anaplastic hobnail cells with clear cytoplasm

A 53-year-old postmenopausal Iranian woman presented to the hospital for a progressively growing painful cesarean scar mass. Her personal history included three previous Pfannenstiel cesarean sections without any medical diseases. The last cesarean section was performed 23 years earlier. Pfannenstiel scars were adjacent to her pubic symphysis. She had no family history of gynecological malignancy. She previously had bloody discharge during her menstrual cycle from a very small orifice inside the abdominal cesarean scar for 16 years. The monthly bloody discharge was stopped after her menopause. She detected a mass that was approximately 3 cm in diameter during her self-examination of her cesarean scar 3 years after menopause. The mass was converted to a huge one over 3 months (Fig. 1). On physical examination, a significant large, firm, and fixed mass was observed in her suprapubic Pfannenstiel cesarean scar and extended upward to the anterior abdominal wall. An abdominopelvic computed tomography (CT) scan revealed a 9.1 × 5.6 × 4.7 cm heterogeneous solid mass with a cystic component in the lower part of the rectus muscle. The observed mass expanded to the subcutaneous tissue, with close contact with the pubic symphysis, and extended to the retropubic space. Other pelvic and abdominal organs were observed to be normal in the first imaging (Fig. 2). Needle biopsy of the mass was performed under ultrasound guidance. Histology revealed an undifferentiated carcinoma. Thorax CT scan, colonoscopy, upper gastrointestinal endoscopy, mammography, and transvaginal ultrasound were all normal. The patient was scheduled for complete excision of the abdominal wall mass as determined by the cancer multidisciplinary team (MDT). A complete excision of the mass and its surrounding tissues was performed. The permanent histology revealed clear-cell carcinoma arising from the Pfannenstiel cesarean scar (Fig. 3). Permanent pathological reports indicated negative excision margins; however, the tumor was very close to the surgical margin in the lower part of the mass adjacent to the pubic symphysis (1 mm).

**Fig. 4:**
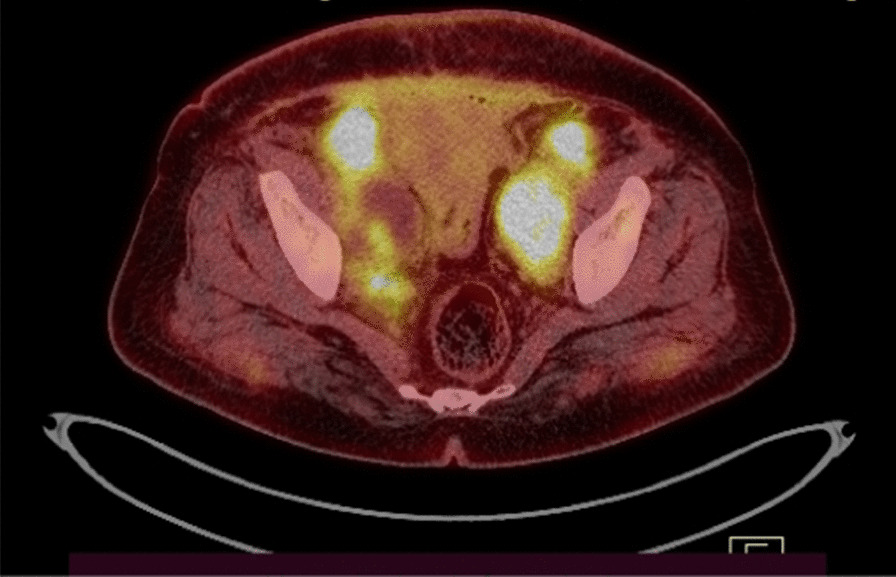
Positron emission tomography–computed tomography 5 months after the first-line chemotherapy. Lower abdominal wall and pelvic peritoneal tumoral involvement

Thirty days after the surgery, the patient initiated chemotherapy with six cycles of paclitaxel and carboplatin every 3 weeks. To follow up, an abdominopelvic ultrasound was performed 3 months after the last chemotherapy treatment. Ultrasound was normal. The patient was asymptomatic nearly 5 months after the termination of chemotherapy. However, she had abdominal discomfort later on. Positron emission tomography–computed tomography (PET-CT) was performed 5 months after the last chemotherapy treatment (Fig. 4) and demonstrated ascites and recurrences in the abdominal wall, retropubic space, and pelvic peritoneum space. Therefore, she received second-line chemotherapy with four cycles of bevacizumab, cisplatin, and doxorubicin. She was resistant to the second-line chemotherapy, and the tumor progressed diffusely in the abdominopelvic space during the second-line chemotherapy course. She had severe abdominal pain and mainly presented gastrointestinal symptoms such as nausea. It was determined by the MDT that the patient was a candidate for palliative therapy. However, she refused the MDT decision and insisted on a second surgery. She had the surgery in another hospital. Her second surgery consisted of total abdominal hysterectomy (TAH) with bilateral salpingo-oophorectomy (BSO), pelvic washing, excision of the abdominal wall mass, segmental resection of a narrow lumen of the large bowel, and end-to-end anastomosis and selective large pelvic lymph node dissection. All specimens showed clear-cell carcinoma involvement. The patient received third-line chemotherapy with three cycles of gemcitabine and vinorelbine. She did not return to our center after the second surgery; however, she sent us her treatment records. Unfortunately, she died 20 months after diagnosis.

### Patient 2

**Fig. 5:**
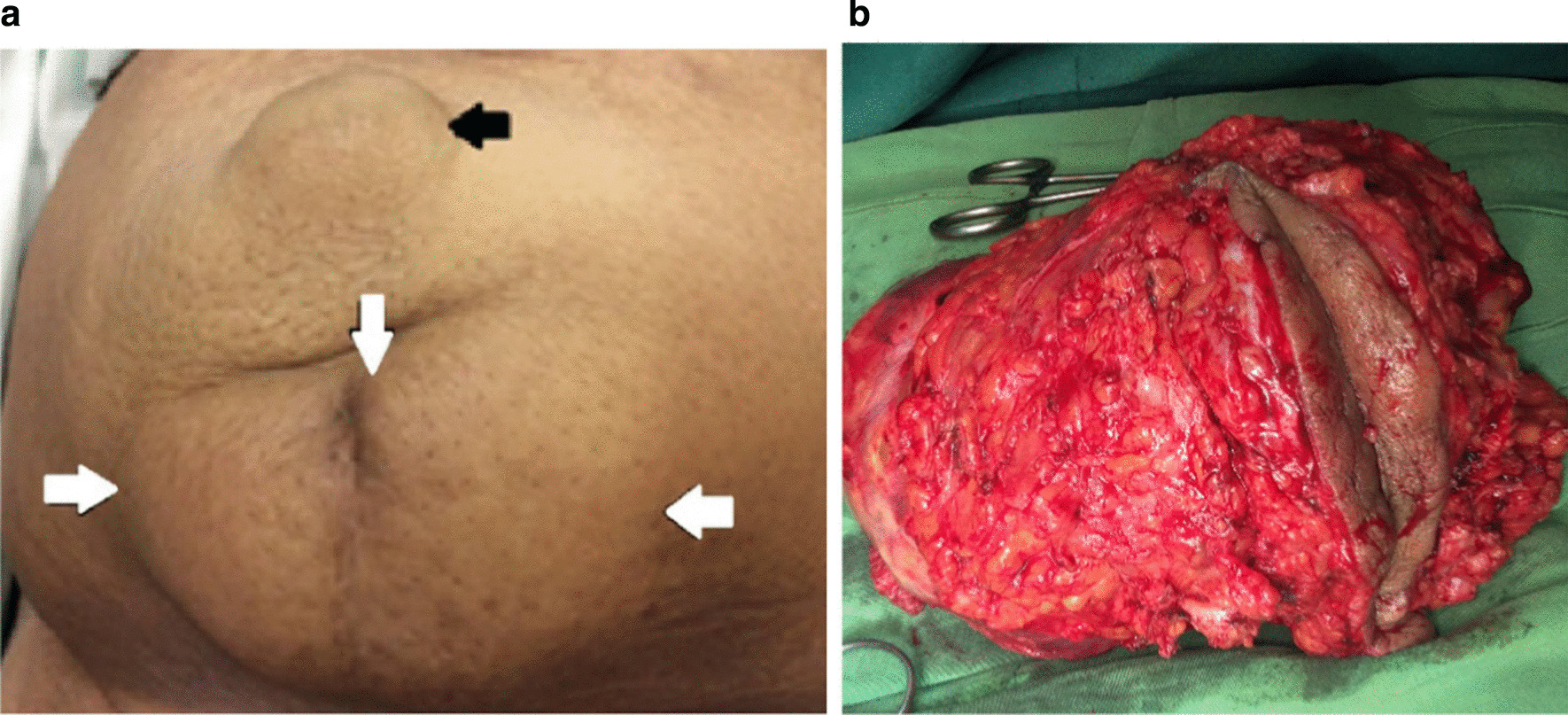
A huge mass originating from the vertical cesarean scar in patient 2. **a** Borders of the abdominal wall mass are marked by white arrows. The black arrow shows the abdominal wall hernia. **b** Total excision of the mass

**Fig. 6:**
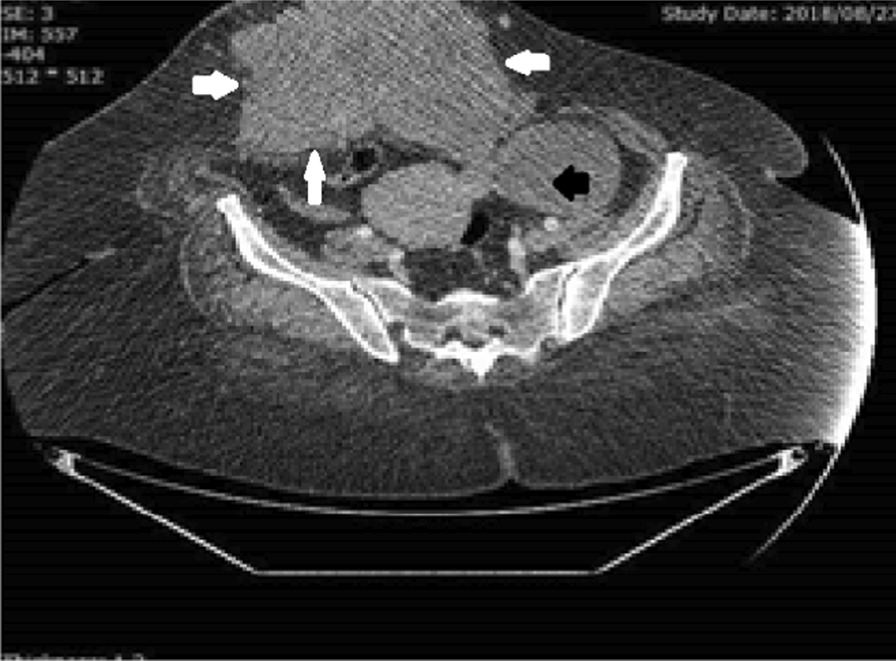
Preoperative pelvic computed tomography scan of patient 2. Borders of the abdominal wall mass are marked by white arrows. The black arrow indicates the pelvic mass

A 58-year-old Iranian postmenopausal woman referred to the hospital because of a abdominal wall mass and hernia. She had a history of three cesarean sections with a midline abdominal incision. Her last cesarean section was performed 20 years earlier. Physical examination demonstrated a partially mobile and large mass in the anterior abdominal wall in the midline cesarean scar, extending bilaterally to the midclavicular line. The upper margin of the mass was adjacent to her umbilicus, where there was a large umbilical hernia above the mass (Fig. 5). Pelvic ultrasound showed an irregular 12 × 8 × 8 cm mass in the abdominal wall anterior to the rectus muscle involving subcutaneous tissue, a large umbilical hernia containing omentum, and a well-defined left solid adnexal mass (4.1 × 3.7 × 5.1 cm in diameter). An abdominopelvic CT scan confirmed the ultrasound findings (Fig. 6). The peritoneal surface of the anterior abdominal wall was intact, and other abdominal and pelvic organs were normal. The chest X-ray was normal.

A local incisional biopsy of the abdominal wall mass was performed before referral to our department. The histology implied clear-cell carcinoma

**Fig. 7:**
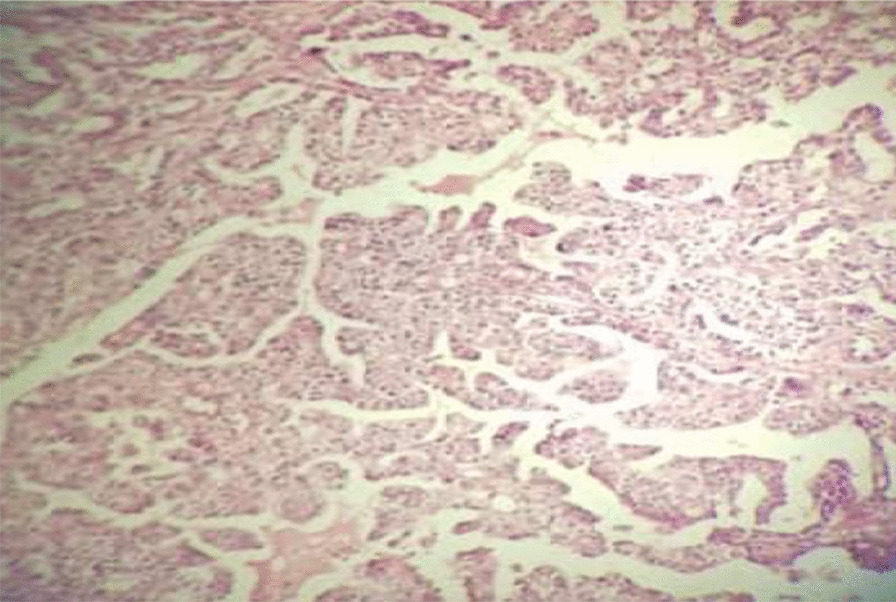
Clear-cell carcinoma of the abdominal scar. Malignant glands are lined by anaplastic hobnail cells with clear cytoplasm

The patient was scheduled for surgery as determined by the MDT. Midline laparotomy was performed. The abdominal wall mass invaded the lower half of the rectus muscles. The mass reported in the left adnexa was indeed a large pelvic lymph node adjacent to the left external iliac artery. Uterus and ovaries had a normal appearance. The patient underwent a complete excision of the abdominal wall mass including the rectus muscles and fascia, peritoneal washing, resection of the enlarged pelvic lymph nodes, TAH, and BSO. Due to the large abdominal wall defect following the excision of the abdominal wall mass, reconstructive surgery was performed to repair the abdominal wall. The defect was covered with a synthetic mesh. The results of the permanent histology of the abdominal wall mass and the enlarged pelvic lymph node were similar, indicating clear-cell carcinoma (Fig. 7) without any marginal involvement. The pathology results of ascites and surgical specimens were negative. Twenty days after the reconstructive surgery, the patient initiated chemotherapy with six cycles of paclitaxel and carboplatin every 3 weeks. An abdominopelvic CT scan was performed 6 months after the completion of the treatment and revealed normal findings. At present, the patient is alive without any recurrence (26 months after diagnosis). The basic characteristics of the patients, imaging results, surgical procedure, and adjuvant treatment are summarized in Table 1.

**Table 1:** Clinicopathological characteristics of two patients with clear-cell carcinoma of abdominal wall cesarean section scars

Characteristics	Patient 1	Patient 2
Age at diagnosis (years)	53	58
BMI (kg/m^2^)	35.4	26.6
Parity	3	3
Tumor size (cm)	9.1 × 5.6 × 4.7	12 × 8 × 8
Type of cesarean abdominal wall incision, no. of times	Pfannenstiel, 3 times	Vertical, 3 times
Symptom	Lower abdominal wall mass	Lower abdominal wall mass
Mobility of mass	Fixed to pubic symphysis	Partially mobile
Time from the last cesarean to disease diagnosis (years)	23	20
Preoperative CT scan results	Lower abdominal wall mass	Lower abdominal wall mass Left solid adnexal mass (lymph node) Umbilical hernia
First surgery	Total abdominal wall mass excision	Total abdominal wall mass excision TAH, BSO, selective large BPLND
Surgical margins of the abdominal wall mass	Free but narrow (1 mm)	Free and wide (more than 1 cm)
Time from the first surgery to recurrence (months)	9.5	No recurrence
Adjuvant chemotherapy, first-line	Taxol + carboplatin 6 cycles	Taxol + carboplatin 6 cycles
Radiotherapy	No	No
Second surgery	TAH+BSO Selective large BPLND Colon surgery (partial obstruction)	No
Current status	Died of disease	Alive without recurrence

*BMI* body mass index, *CT* computed tomography, *TAH* total abdominal hysterectomy, *BSO* bilateral salpingo-oophorectomy, *BPLND* bilateral pelvic lymph node dissection

## Discussion

Clear-cell carcinoma of the abdominal wall that develops in cesarean section scars is an extremely rare condition. Endometriosis of a cesarean section scar or close contact of the abdominal wall with the endometrium of cesarean incision may be a risk factor for the development of abdominal wall clear-cell carcinomas. The incidence of scar endometriosis that is usually the result of a normal endometrial tissue implantation following gynecological or obstetric surgery has been reported to be 0.03–0.4% [8, 9]. The combination of oxidative stress due to inflammation, chronic hemorrhage, and estrogen excess seems to play a role in the tumorigenesis of endometriosis-related neoplasms [9]. Transformation of abdominal wall endometriosis to malignancy is very rare. The malignant transformation of endometriosis within surgical scars is uncommon [10], and the rate of surgical scar endometriosis in the abdominal wall has been estimated to be up to 1% [6, 9].

In the present study, our patients had a history of cesarean section nearly 20 years earlier. The first and second patients had transverse and vertical incisions, respectively. The spreading pattern of tumors was along the direction of the incisions. Transverse spread of tumor along the Pfannenstiel incision scar was observed in the first patient, whereas longitudinal tumor spread along the midline scar was observed in the second patient. These findings are in accordance with the fact that scar endometriosis is an iatrogenic transplantation of endometrial tissues during delivery or gynecological surgery and is not a metaplastic phenomenon [6].

Due to the rarity of abdominal wall endometriosis and transformation of endometriosis to clear-cell carcinoma, the optimal treatment still remains unclear. To date, about 30 abdominal wall clear-cell carcinomas originating from endometriosis cases have been reported in the world literature. Taburiaux *et al*. reviewed 27 cases of cancer arising from abdominal wall endometriosis and reported 18 patients with clear-cell carcinomas [7]. In a retrospective review, Lai *et al*. [11], thoroughly described six cases of pathologically confirmed clear-cell carcinoma originating from the abdominal wall that were encountered at their institution over a 15-year period. They concluded that complete resection of the abdominal wall tumor and suspected intra-abdominal lesions, as well as hysterectomy and bilateral inguinal lymph node dissection, may be suggested as the first step toward its treatment. They recommended adjuvant chemotherapy because of its potential benefits. To achieve complete resection, a surgical procedure such as advanced ovarian cancer surgery may be considered. The surgical planning may include optimal cytoreduction (no residue) and repair of the abdominal wall defect [12].

In our study, only the complete excision of the abdominal wall mass was performed for the first patient at primary surgery, while the second patient underwent TAH, BSO, pelvic washing, and complete excision of the abdominal wall mass and enlarged pelvic lymph nodes. As the first patient had cancer recurrence, perhaps it can be claimed that the difference in surgical treatment was one of the main factors in the recurrence of the clear-cell carcinoma. Hence, it seems that wide local excision of the abdominal wall tumor with TAH and BSO and the exploration of the abdominopelvic space should be preferred during the first surgery. Any imaging is not 100% accurate. Pelvic dissection is also recommended to predict distant metastasis and poor outcome if any positive node is present [9].

Tumor resection with a wide tumor-free margin may be essential for the treatment of an abdominal wall mass. Surgical margins were different in our two patients, as follows: completely negative and wide (more than 1.5 cm) in the second patient and very narrow in the first patient (1 mm). Therefore, we recommend a wide tumor-free surgical margin if possible. It may be that the resection of pubic symphysis during the first surgery of patient 1 resulted in a better prognosis. Moreover, the excision of the lesion adjacent to bone is difficult even in endometriosis of abdominal wall cesarean scars.

These two patients had similar pathology and adjuvant treatments; however, they were different in terms of the direction of tumor spread, proximity to the pubic symphysis, state of margins, exploration of the intraperitoneal space, and performance of TAH and BSO during the first surgery. These differences may have resulted in distinct prognoses for these two patients.

The role of postoperative chemotherapy is not yet well established. Platinum-based chemotherapy has most often been used [6, 9].

## Conclusion

Distinct prognoses were experienced with respect to the clear-cell carcinoma of cesarean section scars presented herein. Based on the findings, complete surgical excision of the abdominal wall mass with wide tumor-free margins, exploration of the abdominopelvic space, TAH, and BSO during the first surgery can be recommended.

To summarize, additional studies are needed to establish a standard treatment in the management of clear-cell carcinoma of cesarean section scars and to benefit from adjuvant treatments.

## Data Availability

All data generated or analyzed during this study are included in the article.

## Abbreviations

TAH: Total abdominal hysterectomy
BSO: Bilateral salpingo-oophorectomy
BPLND: Bilateral pelvic lymph node dissection
CT: Computed tomography
PET-CT: Positron emission tomography–computed tomography
