# Correction to: Clear-cell carcinoma originating from cesarean section scar: two case reports

**DOI:** 10.1186/s13256-021-03035-6

**Published:** 2021-08-10

**Authors:** Seyedeh Razieh Hashemi, Mahdi Morshedi, Houshyar Maghsoudi, Arezoo Esmailzadeh, Ibrahim Alkatout

**Affiliations:** 1 Department of Obstetrics and Gynecology, Baqiyatallah University of Medical Sciences, Tehran, Iran; 2 Department of Surgery, Trauma Research Center, Baqiyatallah University of Medical Sciences, Tehran, Iran; 3 Department of Radiology, Baqiyatallah University of Medical Sciences, Tehran, Iran; 4 Endometriosis Research Center, Iran University of Medical Sciences (IUMS), Tehran, Iran; 5 Kiel School of Gynaecological Endoscopy, University Hospital Schleswig-Holstein, Campus Kiel, Arnold-Heller-Str. 3, Haus 24, 24105 Kiel, Germany

## Correction to: J Med Case Reports (2021) 15:146 https://doi.org/10.1186/s13256-021-02775-9

Following publication of the original article [1], the authors identified an error in the author name of Arezoo Esmailzadeh.

The incorrect author name is: Arezoo Esmaeilzadeh

The correct author name is: Arezoo Esmailzadeh

The author group has been updated above and the original article [1] has been corrected.
